# Medical and Physical Intelligence

**Published:** 1803-03-01

**Authors:** 


### MEDICAL AND PHYSICAL INTELLIGENCE.

### [ FOREIGN AND DOMESTIC. ]

A Cold, attended by symptoms of a very alarming nature, has been general in the city of Paris for some time; it made its appearance anterior to the setting in of any frost, and during the period of heavy rains, which had continued near a month. Women are much more generally affected than men, inasmuch as very few of the latter have been attacked, and these principally old and infirm. Much variety has been observed in the degree of violence of the symptoms, but no particular age, habit, or constitution has been remarked to favour this excess. Some people, extremely delicate, have been but slightly affected, while others, apparently robust and healthy, have fallen a sacrifice. The disease begins by shiverings, violent pain in the head and back; in some instances it has commenced by a violent fit of coughing, and been followed by pain in the head, &c. Though the patient complains much of soreness in the throat, and pain in swallowing, there is in general but little appearance of inflammation on looking into the fauces. The trachea and its lining membrane would seem to be particularly the seat of this soreness; in every case the lungs are more or less engaged, and, in some instances, though not frequently, the person is afflicted by stitches. There is very little expectoration, but the patient labours under a strong desire, and is constantly making efforts to expectorate. The small quantity which is coughed up, is of a purulent nature.

In a great number of cases, the pulse has been but little affected, but in no instance has it been observed to be full, strong, and hard, as in pleurisy or symptomatic fever from inflammation. In those cases where the complaint has been severe, the pulse has been small and quick. In the cases that proved fatal, the lungs were more particularly engaged, and the person died as if from suffocation.

In every instance, the pain in the head has been extremely violent; but a very remarkable symptom, and one which has seldom failed to occur, is a pain in the alveolar processes, extending to the the ears; the cough and thirst are uncommonly troublesome throughout this disorder, which does not seem to have any particular crisis, the symptoms gradually subsiding after an indeterminate number of days; the complaint has sometimes continued three and four weeks. Past experience in the treatment of epidemical complaints of this nature, does not seem to have directed the conduct of the Paris physicians in the treatment of this complaint. In many instances, recourse has been had to bleeding and evacuations. In all the cases that have terminated fatally, this plan had been adopted. Neither emetics nor antimonials seem to be of any service. Sweating, far from being difficult to excite, is very easily produced, but never with any benefit. The disposition to perspire has been remarked very strong in the complaint, and in many instances it has been excessive. In a few cases the complaint has given way, or at least disappeared, after taking a few grains of Dover's powder; and notwithstanding the state of the head and lungs, it has not been observed that opium did any harm; nay, in some instances, it was even useful. The generality of practitioners here have been content with observing the disorder. The use of slops or different kinds of diet drinks were the usual remedies. In those cases where a very opposite practice was pursued, much more benefit was found to arise. When the pulmonary symptoms ran very high, blistering was found of service.

---

Dr. Erdman, of Dresden, in his Medical Observations, relates a curious phenomenon, which was produced by the passion of anger in a boy of a delicate complexion, light hair and a sanguine temper. Whenever this boy fell into a passion, one half of his face would become quite pale, while the other was very red and heated, and these two colours were exactly limited by a line, running down the middle of the forehead, nose, lips and chin. When this boy had heated himself by any violent exercise, the whole face became equally red. It is difficult to account for this phenomenon, which is indeed a remarkable instance of the great influence of passions on the organized body.

---

For preparing the aqua laurocerasi, Van Mons advises to gather the leaves during sun-shine, and having pounded them in a stone mortar, add to the water with which they are to be distilled $\frac{1}{10}$ of brandy, and make a good fire immediately in the beginning of the distillation, because the oil, which has but little volatility, will otherwise not pass over. Professor Wurzer, of Bonn, obtained by distilling one pound of the leaves of laurocerasis with four pints of water, one pint of aqua laurocerasi, which in the dose of fifty drops three times a day, shewed itself very efficacious in hypochondriacal nervous complaints.

---

Dr.

Dr. Henning, of Zerbst, has given an account of a remarkable case of elephantiasis, which he observed in an aged woman. The legs of the patient had begun to swell in her last lying-in, and one of them had broke open, and formed a kind of ulcer. The cause of this complaint was probably venereal. The complaint, against which she had without success employed a great number of remedies, remained almost in the same state till she became fifty years old. About this time an eruption, in form of a thick crust, appeared on the lower part of the leg, which shelling off from time to time, emitted a putrid stinking ichor. The first time Dr. H. saw this affection, was when the patient was near seventy; she was then afflicted with a putrid fever, during which the leg became much worse than it had ever been before; it was dark brown, covered with a thick crust from the sole to the knee, and twice as large as in the natural state. After being recovered from the fever, the patient suffered a great deal of inconvenience from the affected leg: a putrid sanies issued from it, and the itching and pains were almost insupportable; the slightest touch made it bleed, and small ulcers arose at the spots that had thus bled. At last the leg became dry, and the crust shelled off by degrees. Dr. H. applied fomentations of milk with the powder of Peruvian bark, and gave elixir. acid. Haller; but a month after the leg became as bad as before, in which state, having continued for about ten days, it got gradually dry. From this time a most remarkable phenomenon was observed, viz. that the leg got always worse at the appearance of the full moon, and having thus continued for about twelve days, it became gradually better, till the next setting in of the full moon renewed the former state. At last these remissions disappeared, and the leg became no more dry, and continually issued a great quantity of very offensive sanious ichor; the crust was thicker than one inch, and spread itself considerably. The patient was extremely emaciated and weak, had an hectic fever, and being attacked by an affection of the breast, died in the eighty-first year of her age. In the space of thirteen years, during which time Dr. H. had the patient under his care, a great number of different remedies were employed, but without the least success; and among the external remedies, he found a powder of rhubarb and Peruvian bark best calculated for putting a stop to the spreading of this complaint. Dr. H. likewise observed several cases of herpes, in which similar appearances, though in a less degree, occurred; and he is of opinion, that elephantiasis and herpes are very kindred, if not the same diseases, which the climate renders different in their external form. He also thinks that herpes is sometimes communicated by infection, but he never saw an instance where it had disappeared during the period of pregnancy. One case came under his observation, in which an inveterate herpes of the face would disappear as long as the woman was suckling the child, but at the period of ablactation, she had frequent cardialgies, which, having ceased, the eruption appeared again in the face.

Dr.

Dr. Schwaz, of Verden, recommends the following mixture against any obstinate case of taenia: ℞ Petrolei unc. ss. essent. asafoetid. dr. vj. mist. four times a day forty drops. What is most curious, he often tried the ingredients by themselves, but saw no effect except when they were combined.

---

Phosphuret of lime affords a curious compound, on account of the property it has of disengaging, when a few bits of it are thrown into water, a quantity of gaseous bubbles, which, on reaching the surface, inflame spontaneously with a beautiful white flame, and give rise to successive detonations, which may be compared to a running fire of musketry. The method of preparing this substance is as follows: Fill a small glass mattrass, having a flat bottom and long narrow neck, with one part of carbonated lime; place it in a sand-bath, and apply a heat capable of expelling the carbonic acid from the lime. When the decarbonization is near an end, introduce, in portions, a third part of phosphorus, at very small intervals, constantly maintaining the matter at a dark red heat. The phosphorus diffuses itself throughout the whole mass of matter, contracts an union with the lime, loses its volatility, and forms phosphuret. After the whole phosphorus is introduced, let the fire be suddenly slackened, and stop the mattrass with a stopper, having a pneumatic valve, to prevent the access of the air, and which will suffer the gas which remains to escape. When the matter is sufficiently cooled, take it from the mattrass, and put it (taking care not to touch it with the fingers or other moist bodies) into heated glass flasks which can be hermetically heated.

---

From the late important and striking experiments in Galvanism, it appears, 1. That, taking the cessation of excitability to the Galvanic stimulus as the criterion of life, the heart is not the *ultimum*, but the *primum moriens*; for, while the muscles of the limbs were excited to strong contractions, for even several hours after apparent death, the heart was utterly incapable of being excited to action, either by applying the extremity of the metallic arc to the surface or to the interior of this organ. 2. That the lungs were equally inexcitable as the heart. 3. Not only were the muscles, but the skin and cellular membrane, excited by the Galvanic stimulus. 4. The contractions of the muscles were excited by the metallic arc, applied to the nerves supplying the muscles; but the nerves themselves were not affected. 5. The raising up of the arm was produced, as if by volition, by the Galvanic stimulus. 6. A milky or coagulated matter was formed by repeated contractions of the muscle in contact with the copper wire. 7. When the parts ceased to give out motion, the motion was renewed, with augmented force, by wetting them with a solution of sal-ammoniac.

---

Mr. Strenger, of Iver, administers the Galvanic influence, in case of deafness, by applying a small ball to the external orifice of the ear, while a much larger one is held in the patient's hand; hand; the communication is then formed and interrupted alternately by means of machinery, once in every second, for about four minutes daily, for two or three weeks. He asserts that he has thus restored the sense of hearing to forty-five persons.

---

Mr. Cuthbertson has constructed an instrument by which the Galvanic fluid may be applied effectually, for any length of time, without manual assistance, and will, without doubt, hereafter, be as commonly used as our present electrical machines.

---

In comparing Electricity with Galvanism, it must be observed, that the former acts by its intensity, and the latter by its quantity: That the former is sometimes intense enough to strike a man down, and yet not in quantity sufficient to melt a small wire; but the latter will melt metals, and yet scarcely produce a shock.

---

In attempting to restore suspended animation by means of the Galvanic stimulus, it is recommended that oxygen gas should at the same time be applied to the lungs.

---

A very curious and, if true, a most important fact has been reported to the Galvanic Society at Paris, namely, that the fibrine of the blood is sensible to Galvanic irritation, and its contractions become apparent on the application of this fluid.

---

The sensibility with which certain plants appear to be endowed, is it purely mechanical, or has it any analogy with animal sensibility? This question of vegetable physiology, has been the object of a memoir of Cit. Dutrouil, member of the Society of Science, &c. of Bourdeaux. The author first defines the signification of animal irritability; he next examines how far the movements that are perceived in certain plants, when placed in contact with a foreign body, are the indices of an irritability of this kind; he discovers the cause of these movements in the organization of the plant, and explains them in a manner purely mechanical. The author pays particular attention to the sensitive-plant; he attributes the movement which it makes, when touched with the finger, to the action of the electric fluid, and to the sudden disengagement which is produced when put in equilibrium. He confirms this explication by observing that if the plant be touched with a body, which is not proper to transmit the electric fluid, this movement will not take place. Light produces the same effect on the plant as the contact of the finger, by reason of the electricity which is demonstrated to be contained in that agent. The author afterwards attacks the consequence that certain naturalists draw from the approximation of certain parts of the plant at the period of fecundation, (namely that they are endowed with a certain sensibility) by assigning to this approximation a cause purely mechanical; he does not admit in plants the faculty of perception, except it be that of feeling; and he grounds his opinion on the little analogy that there is between their organization and and that of the beings in which this faculty exists, and which they only owe to it.

---

We are happy to inform the public, that the Royal Jennerean Society for the extermination of the Small-pox, is honoured by the patronage of their Majesties and the Royal Family. A great number of the nobility of both sexes have also given it their support, and considerable additions are daily made to the list. The management of the concerns of the Society is referred to three Trustees, a Board of Directors, and a Medical Council. Sub-committees from these are already appointed to provide the necessary accommodations for inoculating in the twelve districts, into which the metropolis is at present divided; in order that the benefits of this invaluable discovery may be diffused as soon as possible. As soon as these are established, due notice will be circulated. As a General Quarterly Meeting of the Society will be held early in March, we hope to be able after that event to announce the commencement of the practice.

---

Dr. Batty's Lectures on Midwifery, and on the Diseases of Women and Children, will commence on Monday, March 21, at his house in Great Marlborough Street.

---

Early in March, Mr. Wagstaffe and Mr. Syer will commence another Course of Lectures on the Elements and Practice of Midwifery and the Diseases of Women and Children, at their lecture room, No. 102, Leadenhall Street.

---

Messrs. Battley and Maitland, of St. Paul's Church Yard, desire us to inform the Public, that the medicine *Lichen Islandicus* may be had genuine at their house.

---

